# Supplementary material for: Developing the digital transformation skills framework: A systematic literature review approach
Source: PLoS One. 2024 Jul 5;19(7):e0304127. doi: 10.1371/journal.pone.0304127 (PMC11226094; doi:10.1371/journal.pone.0304127)
Supplement: S1 Fig — (DOCX) [file pone.0304127.s003.docx]

**S1 Fig. Boolean Search Action used in Web of Science**

((((AB= “digital transformation skill*”) OR (AB= “digital transformation competenc*”)) OR (((AB= “digital skill*”) OR (AB= “digital competenc*”)) AND (AB= “transformation”)) OR (((AB= “digitalization”) OR (AB= “digitalisation”) OR (AB= “digital transformation”)) AND ((AB= “skill*”) OR (AB= “competenc*”))) OR ((AB= “21st-century digital skills”) OR (AB= “21st-century digital competenc*”) OR (AB= “twenty-first century digital skills”) OR (AB= “twenty-first century digital competenc*”)) OR (((AB= “21st-century skills”) OR (AB= “21st-century competenc*”) OR (AB= “twenty-first century skills”) OR (AB= “twenty-first century competenc*”)) AND ((AB= “digitalization”) OR (AB= “digitalisation”) OR (AB= “digital transformation”)))) AND ((AB= “frame*”) OR (AB= “measur*”) OR (AB= “model”) OR (AB= “review”) OR (AB= “instrument”)) AND ((AK= “competenc*”) OR (AK= “skill*”)))
