# Supplementary material for: Developing the digital transformation skills framework: A systematic literature review approach
Source: PLoS One. 2024 Jul 5;19(7):e0304127. doi: 10.1371/journal.pone.0304127 (PMC11226094; doi:10.1371/journal.pone.0304127)
Supplement: Attachment — Submitted filename: DTSF SLR_Plos One_Response to Reviewers.docx [file pone.0304127.s004.docx]

**Authors’ Responses to Reviewer Comments**

Thank you for your feedback and constructive comments. We appreciate the opportunity to revise and resubmit this manuscript. We have made a diligent effort to address each of your comments, and we have responded to each one below. All revisions are noted in the manuscript in red text, and if the revised section is short (a few words, a sentence, or a paragraph), we have included it as part of our response.

| **GENERAL COMMENTS** | |
| --- | --- |
| **COMMENT** | **RESPONSE** |
| 1. When submitting your revision, we need you to address these additional requirements.  Please ensure that your manuscript meets PLOS ONE's style requirements, including those for file naming. The PLOS ONE style templates can be found at  https://journals.plos.org/plosone/s/file?id=wjVg/PLOSOne_formatting_sample_main_body.pdf and  https://journals.plos.org/plosone/s/file?id=ba62/PLOSOne_formatting_sample_title_authors_affiliations.pdf | As requested, we have ensured our manuscript and file names are in compliance with PLOS ONE’s style requirements. |
| 2. Thank you for stating the following financial disclosure: This research was conducted with internal funding provided by the University of Applied Sciences Utrecht.    Please state what role the funders took in the study. If the funders had no role, please state: "The funders had no role in study design, data collection and analysis, decision to publish, or preparation of the manuscript."  Please include this amended Role of Funder statement in your cover letter; we will change the online submission form on your behalf. | The funders had no role in the study. Thank you for providing the appropriate language to use for further clarification. We have revised our Financial Disclosure Statement using the statement provided. Additionally, as requested, we have included this update in the cover letter included with our revision. |
| 3. We note that your Data Availability Statement is currently as follows: All relevant data are within the manuscript and its Supporting Information files.  Please confirm at this time whether or not your submission contains all raw data required to replicate the results of your study. Authors must share the “minimal data set” for their submission. PLOS defines the minimal data set to consist of the data required to replicate all study findings reported in the article, as well as related metadata and methods (<https://journals.plos.org/plosone/s/data-availability#loc-minimal-data-set-definition>).  For example, authors should submit the following data:  - The values behind the means, standard deviations and other measures reported;  - The values used to build graphs;  - The points extracted from images for analysis.  Authors do not need to submit their entire data set if only a portion of the data was used in the reported study.  If your submission does not contain these data, please either upload them as Supporting Information files or deposit them to a stable, public repository and provide us with the relevant URLs, DOIs, or accession numbers. For a list of recommended repositories, please see <https://journals.plos.org/plosone/s/recommended-repositories>. | We confirm that our submission contains all raw data required to replicate the results of our study. In addition to the data already provided in the manuscript (e.g., search terms by abstract and keywords, PRISMA flow chart with inclusion/exclusion criteria, final list of included papers with full citations/DOIs), we have now included the raw datafile of our quality check analysis as Supporting Information (S2 Table). In addition, we also included the exact Boolean search action used in Web of Science (S1 Fig) to increase replicability. As our study is a systematic literature review, there is no other quantitative data to submit. |
| 4. Please review your reference list to ensure that it is complete and correct. If you have cited papers that have been retracted, please include the rationale for doing so in the manuscript text or remove these references and replace them with relevant current references. Any changes to the reference list should be mentioned in the rebuttal letter that accompanies your revised manuscript. If you need to cite a retracted article, indicate the article’s retracted status in the References list and also include a citation and full reference for the retraction notice. | As requested, we have reviewed our reference list for accuracy. We have not cited any retracted papers, nor have we replaced any references. We added one new reference as part of our revision; however, this addition was simply to support clarity of a definition in response to a reviewer comment. We have updated all in-text and reference list numbering accordingly. |
| 5. While revising your submission, please upload your figure files to the Preflight Analysis and Conversion Engine (PACE) digital diagnostic tool, https://pacev2.apexcovantage.com/. PACE helps ensure that figures meet PLOS requirements. To use PACE, you must first register as a user. Registration is free. Then, login and navigate to the UPLOAD tab, where you will find detailed instructions on how to use the tool. If you encounter any issues or have any questions when using PACE, please email PLOS at figures@plos.org. Please note that Supporting Information files do not need this step. | We have completed this step as directed. |
|  | |
| **EDITOR COMMENTS** | |
| **COMMENT** | **RESPONSE** |
| As a tip, please make a list of the criteria used to search the target paper in part of "Search strategy and selection process", such as which keywords are used? | Thank you for your comment. We agree, this is critical information for an SLR. We would like to respectfully note that all of the keywords used in our search process are provided in Figure 2. As part of our revision, we have also provided an expanded version of this figure as Supporting Information which shows all of the variations and combinations of each search term/keyword used:  “For the search action in Web of Science, we combined multiple search terms into one Boolean search operator. Fig 2 presents the search terms by abstract and keywords (see S1 Fig for the detailed Boolean search operator)”. (p. 9, lines 187-188) |
|  | |
| **REVIEWER COMMENTS** | |
| **COMMENT** | **RESPONSE** |
| 1. Is the manuscript technically sound, and do the data support the conclusions?  Reviewer #1: Yes  Reviewer #2: Yes | Thank you. |
| 2. Has the statistical analysis been performed appropriately and rigorously?  Reviewer #1: N/A  Reviewer #2: N/A | Thank you. |
| 3. Have the authors made all data underlying the findings in their manuscript fully available?  Reviewer #1: Yes  Reviewer #2: Yes | Thank you. |
| 4. Is the manuscript presented in an intelligible fashion and written in standard English?  Reviewer #1: Yes  Reviewer #2: Yes | Thank you. |
| 5A. Review Comments from Reviewer #1:  Thank you for the opportunity to review. The manuscript is well written, and the systematic literature search was conducted in line with the recommended guidelines. The methods section is written clearly, and the results are described in great detail. My only minor comments reflect the high quality of this paper. | Thank you so much for your kind comments! We appreciate your constructive feedback and support of our research. We have revised the manuscript in accordance with your comments, as detailed below. |
| In the introduction, it might work better if the authors define Industry 4.0 and digital transformation skills prior to introducing the research questions. | Based on your feedback, we have revised the Introduction to first include an overarching definition of digital transformation, following by a definition of Industry 4.0:  “These advancements drive digital transformation (DT), which comprises the process of improving organizations “by triggering significant changes to its properties through combinations of information, computing, communication, and connectivity technologies” [4, p. 121]. Organizations across sectors undergo DT as they adopt new digital technologies to redefine value propositions for stakeholders as well as optimize or develop digital strategies, structures, processes, and operations [1, 5]. This makes DT a central aspect of Industry 4.0, given that Industry 4.0 entails high digitalization and use of information technologies by organizations to adapt to rapidly changing environments and to gain competitive advantage [6, 7].” (p. 3, lines 53-60)  We also included a definition of digital transformation skills:  “Particularly, employees’ skillsets are often recognized as an important prerequisite to successful DT [8], as advancements of digital technology are shifting the skills needed in the workplace. Employees not only need digital skills, which are the abilities needed to perform and complete job tasks within digitalized work environments [3], but also additional, non-digital, skills to thrive in the context of DT [9].” (p. 3, lines 64-68) |
| Also, it would be beneficial to highlight the gaps in the current literature regarding digital transformation in order to justify the current review. | We also acknowledge the importance of highlighting gaps in the literature better to justify our review and have incorporated this into our Introduction:  “However, there is no consensus on which specific skillsets are needed in the context of DT. Even though there are many scientific articles and models discussing the skills needed for DT, existing frameworks do not cover all the essential skills. This is largely because extant research and frameworks have specifically focused on digital skills [e.g., 10, 11] and, as a result, overlook other crucial skills necessary for exploiting the full potential of DT [9, 12].  Therefore, we attempt to fill this gap in the current literature by making the following three contributions in this research. First, we seek to discern the skills necessary for both the digital technology component and the transformational component of DT, both of which are fundamental in the context of DT [5]. Second, recognizing that DT transcends specific sectors or professions, we pursuit to pinpoint skills that hold relevance across a wide spectrum of professions spanning various sectors. Third, we aim to consolidate the identified skills into a comprehensive framework of DT skills, accompanied by skill definitions to mitigate conceptual ambiguity.” (p. 4, lines 78-89) |
| Under 'Analysis and framework development', it is unclear what the authors meant by 'interrater agreement'. Was it an absolute agreement among raters, and how many raters were involved? | We apologize for the lack of clarity. In all stages which involved interrater agreement, three raters were involved, and absolute agreement was reached. We have clarified this in all relevant Materials and Methods sub-sections:   - p. 9, lines 197-198 - p. 10, line 228 - p. 11, lines 249-250 - p. 12, line 255 |
| 5B. Review Comments from Reviewer #2:  This is a timely paper, it is well researched analysed and presented. It comes at a time when the world, both academic and industry is struggling to make sense of the rapid transformations of work as a result of digitalisation. The framework will therefore make great contribution towards more accurate understanding of industry workforce digital skills needs in order to design effective ways of developing them. The research process and associated arguments are clearly presented in an overall highly accessible writing style. | Thank you so much for your kind comments. We appreciate your feedback and support of our research. |
